# The Natural Whey Starter Used in the Production of Grana Padano and Parmigiano Reggiano PDO Cheeses: A Complex Microbial Community †

**DOI:** 10.3390/microorganisms12122443

**Published:** 2024-11-27

**Authors:** Erasmo Neviani

**Affiliations:** The Italian Committee of the International Dairy Federation, 20131 Milano, Italy; erasmo.neviani@unipr.it

**Keywords:** undefined microbiological complex cultures, natural whey starter, lactic acid bacteria, dairy microbial ecology

## Abstract

Natural whey starter (NWS) is an undefined complex culture used in the production of Grana Padano and Parmigiano Reggiano PDO cheeses. The aim of this review is to discuss, in light of the latest research results, the role of NWS as a primary player in the cheese-making process, considering the microbial community scenario. NWS is traditionally produced by fermenting part of the whey collected at the end of a previous cheese-making process. The method used to produce NWS, based on the back-slopping principle, favors the selection of a microbiota composed mainly of thermophilic lactic acid bacteria. This method of preparation induces the survival of several different species and biotypes. The presence of such a mixture of strains facilitates the development of a natural starter characterized by a remarkable ability to adapt to non-standardized cheese-making parameters. NWS is a microbial community whose activity is not simply the result of the sum of the activities of individual microorganisms, but rather the activity of the community as a whole, in which each individual bacterial cell responds to the presence of the others. According to this traditional protocol, the NWS becomes the ‘microbiological bond’ between cheeses over time.

## 1. Introduction

The capacity of microorganism to colonize an environment includes their ability to interact with other species and biotypes present within the same ecosystem, as well as their capacity to adapt and integrate into the evolving community. The interactions between microorganisms and not just their number, or the presence of different species, biotypes, and variants, in many cases, seems to become a decisive factor for understanding and analyzing the development of microbial ecosystems and the biological function of the individual microbial subjects that are part of them [1,2,3,4,5].

Food fermentation represents an important example of how a microbial community can grow and evolve. In fermented food systems, stimulatory and/or inhibitory interactions between microorganisms can help maintain the viability of a portion of the microbial population and promote the ability of the microbiota to adapt to ongoing frequent and varied changes in the food environment, including those resulting from metabolic activity of the microorganisms themselves [2,3,4,5].

If we consider starter cultures, which are often used to produce fermented foods, we can gain insight into how these microbial communities can cope with the various selective pressures characteristic of food production processes [1,2,3,4,5,6,7,8,9]. For example, in sourdough microbiota, Calabrese and colleagues observed that the microbial communities undergo changes in composition that allow their resilience. For resilience and good performance, the microbial community of sourdough includes dominant and other non-dominant microorganisms, which alternate and work together to ensure good technological performance [2].

The aim of this work is to discuss the role of the natural whey starter (NWS) used for the production of Grana Padano and Parmigiano Reggiano PDO, long-ripened cheeses, as the driver of the cheese-making process, considering the scenario of complex microbial communities and their capability to evolve into the different ecosystem represented by cheese itself and its changes during production and ripening [3,4,5].

## 2. Cheese as a Complex Microbial Ecosystem

Cheese is the product obtained from the acid or enzymatic destabilization of casein, or more commonly from a mixture of both [10,11,12]. The first historical documentation of cheese production is found in ancient texts dating back to around 3200 BC [3,10]. In truth, it is possible that dairy processing of milk spread to different peoples and places around the world even earlier because of chance observations and experiences, later reproduced empirically until it became a defined industrial process through the acquisition of scientific and technological knowledge.

Cheeses are complex biological ecosystems, home to diverse microbial communities. These communities originate from raw milk, starter cultures, and adventitious microorganisms from the dairy environment [3,10,11,12]. 

The great variety of long-ripened cheeses available on the market can also be related to their diverse and distinctive microbiota. The development of the microbiota of long-ripened cheeses involves intricate microbial ecosystems, mainly represented by lactic acid bacteria (LAB), which evolve during the production and ripening of the cheese. 

The contribution of lactic acid bacteria (LAB) to the correct development of the different cheese-making processes developed by man, and to the final quality of cheese, is fundamental. Indeed, the production of most types of cheese involves several modifications of the initial milk components that depend on the LAB metabolic activities [3,9,10,12,13,14,15,16,17]. 

A complex, and only partially understood network of interactions between biotic (e.g., microbial interactions) and abiotic (e.g., technology parameters, pH, aw, redox potential, and chemical composition) factors determines continuous changes in the microbial balance during cheese manufacture [3,10,11,12]. Different conditions adverse to the survival of microorganisms arise in the early stages of cheese making. The technological processes of cheese production imply process parameters that direct fermentation by successively imposing differently selective or elective conditions on the microbiota that follow one another continuously. Furthermore, the environmental conditions and cheese matrix encountered by the microbiota during cheese production are variable, from milk to curd and finally to cheese. This leads to changes in the chemical composition and physical conditions (pH, temperature, and aw) of the growth matrix that influence the microbiota’s ability to adapt and develop [3,10,11,12,14,17]. 

As mentioned above, LAB starters are the main actors in the acidification, first of the milk and then of the curd. Acidification and lactose depletion not only make the substrate less hospitable to most other microbial species but also induce changes in the hydration of casein and its ability to remain in colloidal suspension. The subsequent fermentation of the curd therefore plays a crucial role in helping to define the rheological structure of the future cheese. Indeed, the speed and intensity of acidification cause drastic changes in the stability and structure of casein micelles [10,11,12,18,19,20,21].

At the same time as lactose depletion and the lowering of pH, the mortality of bacterial cells begins to increase dramatically, as does the lysis of LAB starters. Bacterial lysis produces the release of a significant amount of intracellular proteolytic enzymes, both peptidases and proteases. These enzymes can contribute in different ways to the ripening of the cheese, depending on the ripening process and its compositional characteristics, and constitute a kind of ‘inheritance’ of the cheese’s initial microbiota [22,23].

Furthermore, the ability of lactic acid bacteria to grow using cell lysates of other bacteria as a source of nutrients could also be considered an important part of the complicated network of microbial interactions that occurs during cheese ripening [24].

In summary, LAB, first as cellular entities and then as a set of enzymes released after cell lysis, are therefore to be considered the main players in cheese modification [10,11,23].

## 3. Grana Padano and Parmigiano Reggiano Cheeses

Grana Padano (GP) and Parmigiano Reggiano (PR) are two artisanal, traditional PDO cheeses produced in Northern Italy. They are typical, hard, cooked cheeses made from partially skimmed raw milk by acidic fermentation and subjected to slow, long ripening [4,5,12,18,19].

The use of NWS in GP and PR cheese-making was introduced at the end of the 1800s in order to favour curd acidification, and consequently to standardize cheese making process, and to reduce defects of microbiological origin [12,18,19]. 

The NWS is a good example of how humans have learned empirically to use lactic acid fermentation for dairy purposes by leveraging these complex natural microbial ecosystems’ ability to adapt and evolve. NWS microbial community represents the great part of the future microbiota of curd and, in collaboration with the raw milk microbiota, the “engine” of the metabolisms involved in cheese-making and ripening. LAB species and biotypes present in vat milk (coming from NWS and raw milk) may grow, survive, decline, or even become dominant during cheese manufacture. The balance among the microorganisms present in the NWS evolves during cheese making with a gradual process of adaptation to the changing dairy matrix and transferring from the whey to the vat milk and finally to the curd [4,5]. 

**Figure 1:**
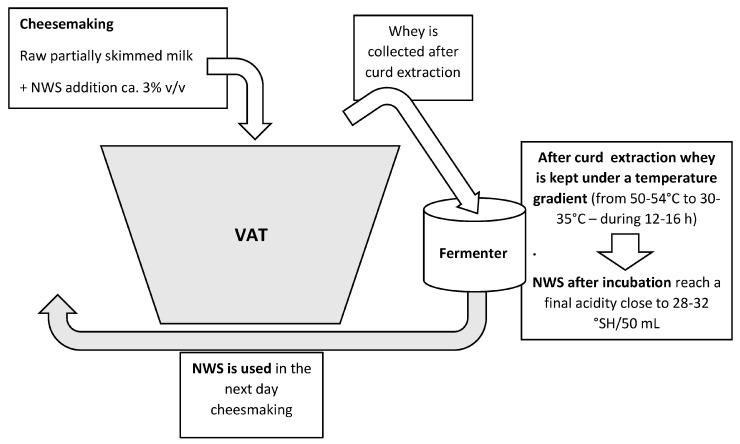
NWS cyclic production process based on back-slopping.

NWS is a natural starter traditionally prepared by leaving part of the whey, collected after the separation of the curd, to ferment in conditions of particular thermal gradient (Figure 1). After fermentation, NWS reaches an acidity of around 30 °SH/50 mL and a pH close to 3.5. NWS is added to cow’s milk, which is then heated to 32–34 °C and coagulated with powdered calf rennet. When the curd has reached the right consistency, it is broken down into very small granules, mixed for 5–15 min, and cooked by heating at 53–56 °C. During cooking, the acidifying activity of the lactic bacteria favors the formation of the correct rheology of the curd. Once stirring is complete, the curd granules settle on the bottom of the tank, where they aggregate under the whey for approximately 30–50 min, at a temperature no higher than that reached at the end of cooking. Under these conditions, thermophilic lactobacilli grow and become the predominant microbial population in the acidifying curd.

The NWS lactic acid bacteria that colonize the curd matrix and freshly produced cheese reach population values close to the hundreds of millions of cfu/gr. These high numbers of LAB cells and the enzymes released by their lysis play a large role in cheese ripening [4,5,22,23,24]. Gatti and colleagues [5] extensively discussed the evolution of the microbiota of Grana cheese during production and ripening. 

## 4. Natural Whey Starter—A Peculiar Complex Microbial Ecosystems

As previously introduced, the NWS preparation method favors the presence in the acidified whey ecosystem of a large number of different microorganisms, in particular LAB originated from the vat milk microbiota (mixture from raw milk and NWS microbiota). The NWS microbiota turns out to cope with the selection imposed by the different technological parameters involved in cheese-making and its preparation from sweet whey. The NWS is produced by incubating part of the “sweet” whey collected in the previous cheese-making, after curd breaking and cooking (Figure 1). After extraction from the vat, the whey is kept under a temperature gradient (from ca. 50–54 °C to about 30–35 °C—12–16 h) to reach a final acidity close to ca. 28–32 °SH/50 mL. The temperature reached by cooking the curd is maintained for a significant amount of time, and the cooling of the extracted whey is slow and gradual. The natural temperature gradient that develops during the fermentation of the sweet whey and the increase in its acidity induce the selection of a characteristic thermophilic and acidophilic microbiota [3,4,5,12].

Because of its production method, natural whey starter cultures result in undefined, multiple-strain culture communities of a great amount of thermophilic LAB, in association with minor amounts of mesophilic LAB [4,5,25,26,27,28,29,30]. The resulting natural whey starter microbial composition is dominated by thermophilic lactic acid bacteria such as *Lactobacillus helveticus*, *Lactobacillus delbrueckii* subsp. *lactis*, *Lactobacillus delbrueckii* subsp. *bulgaricus, Streptococcus thermophilus*, and *Limosilactobacillus fermentum.* Homofermenting LAB species largely prevail against heterofermenting ones [4,5,9,25,26,27,28,29,30,31,32].

A large amount of the natural whey culture (ca. 3% *v*/*v*) is added to the vat milk [4]. 

The peculiar adaptive characteristics of the main thermophilic species of NWS allow this natural culture to maintain its technological function by adapting to a cyclic production process based on back-slopping (Figure 1). Among the resilient bacteria are numerous biotypes that will be important in the stages of curd acidification and, later, in the early ripening process [3,4,5,12]. 

In order to understand its overall resilience, linked to the presence of different thermophilic LAB biotypes, it is important to emphasize that the bacterial cells in the natural whey select themselves in reaction to two different temperature gradients that they undergo and that expose them to particular thermal stresses: initially, after inoculation into the milk, the temperature increases to 52–54 °C, while after separation of the whey from the curd, the temperature of the natural culture decreases naturally and slowly, either through exposure to room temperature or through incubation in the fermenter. In summary, the main factors determining the selection of lactic acid bacteria are (i) high incubation temperature (in a temperature gradient), (ii) low pH at the end of incubation (below 4.0), and (iii) back-slopping due to milk inoculation [4,5,27].

The two main thermophilic species in NWS, *L. helveticus* and *L. delbrueckii* subsp. *lactis*, seem to respond differently to the two gradients and to the different composition of the environment during the production phases. Indeed, *L. helveticus* is considered a more acid-tolerant strain, and this could explain why this strain could increase in numbers after overnight incubation of natural starter whey, reaching lower pH values. In contrast, inoculated vat milk has higher pH values, which, in combination with a possible greater tolerance to thermal stress of *L*. *delbrueck*ii subsp. *lactis*, could explain the numerical increase observed after the curd-cooking step [4,5,27,32].

As pointed out above, the preparation method ensures the survival in NWS of many different biotypes, and perhaps variants, of the two majority thermophilic LAB species. The presence in the NWS of different biotypes of the same species has been verified and discussed in several previous works. In particular, differences were highlighted at the genotypic level and in the expression of some relevant phenotypes (e.g., enzymatic activities, resistance to phage infections) [3,33,34,35,36,37]. The presence of a mixture of different biotypes of the same species facilitates the development of an NWS characterized by a poorly defined microbial composition but with a strong adaptive capacity [5,27]. In previous works it was possible to highlight how the microbiological composition of the NWS differed in relation to the technological peculiarities of the different dairies and how it adapted over time to possible and inevitable environmental and technological modifications [3,4,5,33,34,35,36]. A change in the dominant biotypes in several NWS over time has also been observed in recent decades. This aspect favors its technological functionality in artisanal cheese-making processes. In essence, it is a selection that follows Darwin’s principles, i.e., only biotypes with a better aptitude for adapting to hostile environmental conditions survive and persist in the ecosystem. The presence of different LAB species and biotypes is a crucial aspect that helps us to distinguish natural starters from selected starters. 

In numerous studies it was possible to observe how a significant part of the biotypes present in the NWS were not able to survive alone when placed to grow in pure cultures after isolation. By comparing culture-independent and culture-dependent enumerations of NWS populations [28,29,38], the number of total cells, and in particular viable cells, is often higher (up to 1 log unit) than the number of cultivable cells, evaluated as colony-forming units. Comparing cultural and non-cultural methods to evaluate the evolution of the lactic acid microbial load from whey to mature cheese, the presence of a high number of viable non-cultivable bacteria (VBNC) was confirmed. During ripening of the cheese, the gap of values obtained comparing VBNC and colturable bacteria decreased, both in absolute value and in percentage [38]. From this observation, one could draw the conclusion that a part of the cheese microbiota, coming from the whey, is able to grow only in the specific ecosystem (whey, curd, or cheese) while it is poorly adapted to growth in different synthetic media, despite their rich nutritional composition. Two effects add up to explain this evidence: the first is a positive effect connected to the compositional characteristics of the matrix (abiotic interaction), and the second is the effect of synergistic interaction between bacteria present in the characteristic microbial ecosystem (biotic interaction). Giraffa and colleagues showed in an in vitro study numerous potential bacterial interactions, with both stimulatory and inhibitory effects, between biotypes belonging to the species *L. helveticus*, *L. delbrueckii* ssp. *Lactis*, and *L. fermentum* [7]. The interactions observed were evident already between simple pairs of biotypes, and this would lead one to think that complex networks of interactions could be established in a system inhabited by numerous microorganisms.

Considering the reduction of the delta between the values of the microbial population obtained by cultural and non-cultural analyses, observed during cheese ripening [33], one might suppose that if the total number of bacteria decreases, then the interaction useful to survive in the microbial ecosystem for non-culturable cells also decreases. In other words, we can conclude that the presence of a high number of bacteria in a microbial ecosystem also favors the survival of bacterial cells incapable of developing alone in the specific matrix and which require synergistic interactions with culturable bacteria. Reducing the overall population reduces this phenomenon and also results in a reduction of non-culturable cells.

Puzzles and doubts remain about the real role of VBNC LAB present in a dairy ecosystem or food ecosystem in general. These are often a majority part of the microbial population present in a system (NWS, curd, and cheese). These bacteria appear unable to grow in different selective culture media and therefore cannot be isolated and studied. As a result, the role of this part of the population present in the NWS and in cheese is not currently known. However, we must remember that the biological significance of VBNC cells and their survival mechanisms have yet to be studied, and the VBNC condition is controversial [39].

One of the advantages observed for NWS biological systems is their wide resistance to lytic bacteriophage attacks. The consistent presence of lytic phages in the NWS did not impair their performance, and this could be related to the presence of various bacterial biotypes, with different phage sensitivity profiles, allowing them to effectively counteract the phage predation [40,41,42]. The presence of a bacteriophage favors the development of phage-resistant biotypes that take over the culture and which, in turn, will later be replaced by new biotypes resistant to future phage infections. The presence of phage-resistant biotypes leads to the decrease and disappearance of certain bacteriophages, which are no longer able to multiply. It is, in effect, a system that has considerable resilience and can only be interrupted in the presence of numerous and simultaneous phage infections.

We can therefore summarize that the NWS microbial complexity is useful for the development in different abiotic conditions of the ecosystem itself and its ability to self-adapt to the environmental conditions. Interaction among the different strains present in the ecosystem, regardless of their number and their state of viability, can be relevant in defining the metabolic activity of the natural whey ecosystem as a whole [4,5,27,28,29].

If we try to simplify the composition of the NWS microbiota, we can observe many different microorganisms that we can arbitrarily separate into three groups [5]. A large proportion of the microorganisms seem to be present only occasionally and in variable numbers in the NWS. This first group (part A) is larger and variable, probably also in relation to the use of raw milk, as in the case of GP and PR cheeses. These are microorganisms probably without great technological significance or with minor functions not yet understood. The subsequent passages of the whey in raw milk (production based on the back-slopping procedure) also favor the dragging of these simply contaminating microorganisms from the milk to the NWS. A second part of the microbiota (part B) consists of microorganisms (biotypes and perhaps variants) that are functional and necessary to the survival of the NWS ecosystem itself. This is the part of the microbiota that is necessary for speedy and correct acidification of the whey, which determines the resilience of the whey starter ecosystem as a whole and represents the component that determines the continuity and survival of the ecosystem. There is a third part of the NWS microbiota, a minority part, which represents the microbiota necessary for the fermentation of curd, but which at the same time is not strictly necessary for the fermentation of “sweet” whey at NWS (back-slopping). It can be hypothesized that this third part (part C) represents the microbial nucleus mainly responsible for the process of transforming fresh curd into cheese. Unlike part B, part C does not represent the biological core of stability and continuity of the NWS ecosystem in itself. In fact, using this part of the microbiota alone, unlike the case of part B, it would not be possible to reproduce the functionality and complexity of the NWS.

The division and segregation of parts of the NWS microbiota, proposed above, is obviously an excessive simplification, useful for understanding the different roles that different components of the NWS microbiota can play as a priority in the context of cheese making and ripening. It is likely that the different parts of the NWS microbiota (A, B, and C), or at least some of the microorganisms that compose them, are useful from an ecological point of view as a whole and interact in a network of complex metabolic relationships. 

In a recent work on NWS to produce Parmigiano Reggiano cheese, Bertani and colleagues [27] observed a core microbiota adapting, in turn, to a mixture raw milk and whey (not acidified whey) or acidified whey. Rossetti and colleagues highlighted the majority presence in curds of certain biotypes present as minorities in the NWS [25,31]. Most of the biotypes present in the NWS are those that are best suited to its preparation by fermentation. However, there are also minority biotypes capable of adapting and developing in the milk in the vat or in the curd environment. This last minority part of the natural microbiota of whey could be considered the essential one for the correct conduct of the cheese-making process. In a certain sense one could suppose that this minority part survives in the NWS because it is somehow protected and helped by the majority of the microbial population.

For resilience and good performance, the natural whey starter community seems to include dominant and non-dominant players that can change their role by adapting to changes in matrices. The presence of several microbial components of the complex ecosystem that are important for its technological activity would explain the difficulty of replacing the natural culture with the selected one. In fact, it does not seem to be sufficient to use mixtures of strains isolated from milk or from natural starters to obtain starters with good cheese-making performance. It would rather be necessary to identify, isolate, and select from the NWS also those minority biotypes that are characterized by good adaptation and development capabilities in the cheese curd. In any case, in order to reproduce the activity of the NWS ecosystem it may be essential to reproduce the cellular interactions that characterize it. To this end, minority and undervalued parts of the population could be useful for the technological performance of the starter or even apparently non-viable strains.

## 5. Microbial Interaction and Complexity in Natural Consortia: NWS as a Model for Functional Microbial Ecosystem

Complex microbial communities exist everywhere in nature, and the interactions between microorganisms, which make up the community, are a crucial aspect that conditions their development. Each individual cell of a complex population responds to the presence of others in the consortium. The possible division of metabolic work among the members of the microbial community produces an overall result that exceeds the simple sum of the work of the individual subjects of the community and which can only be explained by combining and integrating the tasks performed by single individuals. These interactions play a very important role in the evolution of partners and allow multiple microorganisms to survive even in the presence of limited nutritional resources [43,44,45,46,47,48,49]. Microbial consortia rapidly adapt to changes in their environment, and, at the same time, microbial populations can improve their performance thanks to the contribution of individual mutations [1,50,51,52,53]. The interaction with the environmental matrix can also be essential in defining the evolution and development of microbial communities [54,55,56].

The hypothesis of thinking of complex microbial ecosystems as multicellular organisms, whose individual components interact and influence each other [48,49], remains highly suggestive. In general, it can be stated that complex microbial consortia perform more complex activities (versatility) and tolerate more variation in the environment (robustness) as compared to pure cultures [47,48,56]. Taking this scenario into account, the different types of interactions that occur between microorganisms, both cooperation and competition, and not just their number or the presence of different species, biotypes, and perhaps variants, become the determining aspect for understanding how we can develop complex microbial ecosystems [43,56,57,58,59,60].

The interactions between microorganisms become the decisive aspect that conditions the development of NWS. The NWS community therefore seems to include main and less central actors, protagonists and extras, who can alternatively take on priority roles by adapting to changes in the ecosystem. The microbial complexity observed in the NWS for GP and PR probably is not exclusive to this type of natural starter and can also be observed in other natural systems involved in food production [3,6]. The peculiar way of preparing the NWS, which follows the back-slopping procedure, enhances the development and functionality of this complexity. Consequently, complex communities like NWS must be studied in terms of function rather than composition. In this context, populations are to be understood as collectives that become the fundamental unit of life [56,57,58,59,60]. We can think of the NWS as a specialized microbial social structure, whose members adapt, from time to time, to colonize in a complementary and strategically orchestrated way a changing dairy environment that modifies in relation to the technological parameters used by the different dairies. 

Complex microbial populations, such as NWS, appear to function as minimal fundamental units necessary for understanding their functional and technological roles.They are ecosystems in which most interactions take on a physiological and metabolic significance that is essential for the survival of the community itself. The single microorganism is effectively trapped in deep networks of interdependence, both evolutionary and ecological [59].

Following this interpretation, the relevance of the presence of different strains or biotypes in the NWS could not be understood by merely studying the pure culture of its individual constituent strains in detail. The presence of several components of the complex ecosystem that are important for the activity of the ecosystem would explain the difficulty of replacing the natural culture with the selected one. The understanding of how these natural communities model themselves and behave remains a decisive step in being able to hypothesize reproducing their attitudes in selected starter cultures.

## 6. Conclusions

Despite the important results provided by previous studies, the secrets of NWS dairy efficiency are not yet fully understood. To achieve this goal, I think that further investigations will be necessary, both on the metabolic potential of individual strains and biotypes and also, perhaps above all, on how complex communities behave. Following this approach, the subject of evolution is no longer the individual but becomes the community. 

After dedicating extensive time and effort to understanding the individuality of the single microbial cells and their mechanisms of surviving, perhaps it is time for a journey backwards from the single bacterial cell to the community, “from small to the complex”. Journey, which will be necessary for a better understanding of microbial complex ecosystems functionality and their importance for our life.
